# The influence of heat stress on auxin distribution in transgenic *B. napus* microspores and microspore-derived embryos

**DOI:** 10.1007/s00709-014-0616-1

**Published:** 2014-02-20

**Authors:** Ewa Dubas, Jana Moravčíková, Jana Libantová, Ildikó Matušíková, Eva Benková, Iwona Żur, Monika Krzewska

**Affiliations:** 1 The Franciszek Górski Institute of Plant Physiology, Polish Academy of Sciences, Niezapominajek 21, 30-239 Kraków, Poland; 2 Institute of Plant Genetics and Biotechnology, Slovak Academy of Sciences, Akademicka 2, P.O.B. 39A, 95 007 Nitra 1, Slovak Republic; 3 Department of Plant Systems Biology, VIB, Gent University, Ghent, Belgium; 4 Institute of Science and Technology Austria (IST Austria), Am Campus 1, 3400 Klosterneuburg, Austria

**Keywords:** *Agrobacterium tumefaciens*, Auxin, *Brassica napus*, DR5 promoter, DR5rev promoter, *gfp* gene, *gus* gene, Microspores, Microspore-derived embryos, Polarity

## Abstract

Plant embryogenesis is regulated by differential distribution of the plant hormone auxin. However, the cells establishing these gradients during microspore embryogenesis remain to be identified. For the first time, we describe, using the DR5 or DR5rev reporter gene systems, the GFP- and GUS-based auxin biosensors to monitor auxin during *Brassica napus* androgenesis at cellular resolution in the initial stages. Our study provides evidence that the distribution of auxin changes during embryo development and depends on the temperature-inducible in vitro culture conditions. For this, microspores (mcs) were induced to embryogenesis by heat treatment and then subjected to genetic modification via *Agrobacterium tumefaciens*. The duration of high temperature treatment had a significant influence on auxin distribution in isolated and in vitro*-*cultured microspores and on microspore-derived embryo development. In the “mild” heat-treated (1 day at 32 °C) mcs, auxin localized in a polar way already at the uni-nucleate microspore, which was critical for the initiation of embryos with suspensor-like structure. Assuming a mean mcs radius of 20 μm, endogenous auxin content in a single cell corresponded to concentration of 1.01 μM. In mcs subjected to a prolonged heat (5 days at 32 °C), although auxin concentration increased dozen times, auxin polarization was set up at a few-celled pro-embryos without suspensor. Those embryos were enclosed in the outer wall called the exine. The exine rupture was accompanied by the auxin gradient polarization. Relative quantitative estimation of auxin, using time-lapse imaging, revealed that primordia possess up to 1.3-fold higher amounts than those found in the root apices of transgenic MDEs in the presence of exogenous auxin. Our results show, for the first time, which concentration of endogenous auxin coincides with the first cell division and how the high temperature interplays with auxin, by what affects delay early establishing microspore polarity. Moreover, we present how the local auxin accumulation demonstrates the apical–basal axis formation of the androgenic embryo and directs the axiality of the adult haploid plant.

## Introduction

The plant hormone auxin (indole-3-acetic acid, IAA) has been identified as a factor controlling major cell specific events with the process of embryo formation and differentiation (Möller and Weijers 2009), root patterning (Overvoorde et al. 2010), organ formation (Sundberg and Østergaard 2009), vascular tissue differentiation (Scarpella et al. 2006), and growth responses to environmental stimuli (Halliday et al. 2009).

Several methods have been successfully applied to monitor auxin distribution including immunolocalization (Benková et al. 2003), direct measurements of endogenous auxin (Friml et al. 2002; Dubas et al. 2012a, b; 2013a, b), radioactive labeling (Rashotte et al. 2000), or indirect determination of the activity of auxin-responsive gene promoters (Friml et al. 2003).

The finding of the consensus TGTCT (AuxRE) sequence within the promoter region of auxin-responsive genes such as *Aux/IAA*, *GH3*, or *SAUR* (see review Hagen and Guilfoyle 2002) enabled to prepare synthetic, highly auxin-inducible promoters such as DR5 and its variant DR5rev. They are improved derivatives of the promoter of the soybean *GH3* gene. The promoters contain multiple AuxRE repeats modified with site-directed mutations (Ulmasov et al. 1997). Using the DR5-reporter gene system, the auxin distribution and transport have been successfully studied mainly in *Arabidopsis thaliana*, during zygotic embryogenesis (Benková et al. 2003; Friml et al. 2003; Sauer and Friml 2004; Jenik and Barton 2005; Weijers and Jürgens 2005), floral development (Aloni et al. 2006), and in roots (Jones et al. 2009), seeds, and cotyledons of young seedlings (Nakamura et al. 2003; Li et al. 2009). Only a few experiments have been performed on other plant species such as *Physcomitrella patens* (Bierfreund et al. 2003), *Nicotiana tabacum* L. (Chen et al. 2010), or *Cucurbita pepo* (Ilina et al. 2012).

Embryogenesis is one of the most important processes in developmental biology. A polar pattern of auxin distribution is crucial for appropriate embryo development *in planta*. Studies on *Arabidopsis* zygotic embryos showed that auxin is distributed directionally throughout the embryo from the places of its synthesis and that the polar auxin transport is mediated by auxin influx and efflux facilitators. The DR5 promoter activity changed dynamically in asymmetric distribution patterns, starting from the two-cell pro-embryo to the 32-cell-embryo stage with auxin maxima in the apical cells and its descendants. At later stages, the highest activity characterized the uppermost suspensor cells, including the precursor to the root meristem—the hypophysis, the cotyledon apices, and the provascular tissue (Friml et al. 2003).

On the contrary, in microspore embryogenesis, the occurrence of a polar auxin distribution pattern has to be assigned. *Brassica napus* microspore suspension can serve as a perfect model system to study the role of auxin in the early phases of microspore-derived embryo (MDE) initiation and differentiation without any sporophytic tissue interference. However, a wider application of molecular tools such as a genetic modification is still hampered by the genotype specificity of the microspore embryogenesis response and/or poor regeneration potential of produced embryos (Datta 2005). Moreover, there are only a few papers reporting successful *Agrobacterium*-mediated transformation of *B. napus* microspores (mcs; Abdollahi et al. 2009) or MDEs (Cegielska-Taras et al. 2008).

Due to the fact that, at the uni-nucleated and early bi-nucleated phases of development, mcs are still totipotent, they can be reprogrammed to form embryos under specific in vitro culture conditions. Stress was identified as the main trigger that initiated this developmental switch, whereas many other factors including “donor plant” genotype and its physiological status, microspore developmental stage, and culture conditions significantly influenced the effectiveness of the process (Wędzony et al. 2009). It has been revealed that in the case of *B. napus*, heat stress is the most effective stressor and that the pattern of embryogenic development is highly dependent on the intensity and duration of this treatment (Zhao et al. 2003). The “mild” heat stress (1 day at 32 °C) triggers mcs to form embryos with a suspensor-like structure that highly imitates zygotic embryos at the successive developmental stages. The prolonged heat stress (5 days at 32 °C) induces symmetrical divisions within the exine leading to conventional microspore cultures with multicellular, suspensor-deprived embryogenic structures (Custers et al. 1994; Hause et al. 1994; Yeung et al. 1996; Joosen et al. 2007; Supena et al. 2008; Dubas et al. 2011; Dubas et al. *in preparation*).

In this study, heat-induced microspore cultures of *B. napus* were subjected to genetic modification via *Agrobacterium tumefaciens.* Transformed mcs and MDEs containing either the reporter β-glucuronidase (*gus*) or the green fluorescent protein (*gfp*) markers under control of the synthetic auxin-responsive DR5 or DR5rev promoters (respectively) were used to study auxin distribution at the early stages of microspore embryogenesis. To our knowledge, there are no reports examining the effect of heat stress treatment on the early regulation of auxin maxima in embryogenic suspensions of cells without any sporophytic tissue interference. For the first time, using the DR5 reporter gene systems, we were able to present the moment when the early embryo polarity started. The transformation to asymmetrically localized auxin was regulated at the few-celled pro-embryo when exine ruptured. To date, it has been difficult to model the progression from symmetry to asymmetry in plants starting from the single cell.

## Material and methods

### Vector constructs and bacterial strains

The plasmid pUC19 DR5-GUS (kindly provided by Prof. T. Guilfoyle, Dept. of Biochemistry, University of Missouri, Columbia) contained the *gus* reporter gene under control of the synthetic auxin-responsive DR5 promoter. To prepare plant transformation vector pDR5::GUS, the sequence of *DR5/gus/nosT* was cloned as *Sal*I-*Eco*RI fragment from pUC19 DR5-GUS into the binary vector pBinPlus (Van Engelen et al. 1995).

The plant transformation vector pDR5rev-SV40-3xGFP (pDR5rev::GFP), a derivate of binary vector pGreenII, was kindly provided by Dr. Weijers (Laboratory of Biochemistry, Wageningen University, The Netherlands). This vector contains the synthetic auxin-responsive DR5rev promoter driving the expression of GFP marker by incorporating a nuclear localization signal.

The binary vectors pDR5::GUS and pDR5rev::GFP were introduced into *A. tumefaciens* strains LBA 4404 and GV3101, respectively.

Bacterial inoculum was prepared from the overnight culture of *A. tumefaciens* LBA 4404/pDR5::GUS or *A. tumefaciens* GV3101/pDR5rev::GFP (individually). Bacterial cells were pelleted by centrifugation and re-suspended in NLN-13 medium containing 0.01 M D-glucose to optical density OD_600_ = 0.6.

### Microspore preparation and transformation

Mcs were isolated from *B. napus* L. cv. Topas line DH 4079 according to the protocol by Custers (2003) with modifications by Joosen et al. (2007). Mcs were cultured in Petri dishes at the density of 40,000 cells per 10^−3^ L in NLN medium with 13 % sucrose (NLN-13; Lichter 1982). Embryogenic cultures were obtained by applying (1) 32 ± 0.2 °C for 5 days and thereafter 25 °C in darkness (prolonged heat shock) or (2) 32 ± 0.2 °C for 24 h and thereafter 25 °C (“mild” heat shock). For transformation experiments, microspore suspension cultures (7 or 21 days post heat stress) were centrifuged, re-suspended in NLN-13 medium supplemented with 0.01 M D-glucose, and co-cultivated with the same volume of bacterial inoculum. The co-cultivation medium was supplemented with 20 μM of acetosyringone. Following 2 days of co-cultivation at 22 °C in darkness, the mcs were washed three times using NLN-13 medium supplemented with 500 mg L^−1^ cefotaxime. Then, the microspore suspension was continuously kept in NLN-13 medium supplemented with 100 mg L^−1^ cefotaxime.

### Histochemical GUS assay

Histochemical GUS assay was carried out according to the method of Jefferson (1987). Mcs and MDEs were submerged in the GUS staining buffer containing 2 mM 5-bromo-4-chloro-3-indolyl-β-D-glucuronide (X-Gluc) and 50 mM sodium phosphate buffer (pH 7), vacuum infiltrated for 10 min at room temperature, and incubated at 37 °C in the dark for 24 h. The samples were whole mounted on microscope slides in a clearing solution of chloral-hydrate:glycerol:water (8:1:2 [v:v:v]) as described by Berleth and Jürgens (1993) and analyzed under Nikon Eclipse E-600 microscope with Nomarski differential interference contrast (DIC) system. Images were acquired and processed using software programs including NIS Elements AR software (Nikon) and CorelDRAW^®^ ESSENTIALS.

### GFP expression analysis and the relative fluorescence intensity measurements

GFP expression analysis was performed at 10–30 min intervals over 250 min with non-immobilized cultures that were left in dark on a fluorescence stereo-microscope (Leica MZ10 F; Ex/Em 470/40 nm, DM 495 nm BF 525/50 nm) equipped with digital camera (Leica DFC 420C). In order to quantify the expression of GFP fluorescence in the MDEs, suspension with MDEs was exposed to exogenous auxin (indole-3-butyric acid (IBA), 1 mg L^−1^). The relative fluorescence intensity (RFI) of DR5rev::GFP was measured in individual MDEs in a period of 220 min (30–250 min). Interval 0–30 min was dedicated for control (culture without the presence of exogenous auxin). To estimate the RFI, gray levels across single regions of MDEs (including root primordial and the root tip) were quantified using the base package of ImageJ software. RFI was measured three times for population of 100 transformed MDEs after 1 month from the inoculation.

### Estimation of endogenous auxin level

Endogenous auxin was measured in mcs induced to embryogenesis by heat (32 °C) treatment for 1 or 5 days. The detailed procedure for auxin extraction, purification, and quantification by high-performance liquid chromatography (HPLC) was described by Dobrev and Kamínek (2002) and Dobrev et al. (2005). Harvested mcs were immediately frozen in liquid nitrogen. Material was collected at least in three biological replicates.

Auxin concentration (μM) in mcs was estimated according to the mean diameter of mcs (10 or 20 μm). Mcs’ diameter was measured three times for a population of 500 msc collected directly after isolation or after 1 day treatment at 32 °C. Measurements were done under a Nikon Eclipse E-600 light microscope equipped with NIS Elements AR software (Nikon).

## Results and discussion

*Agrobacterium*-mediated DNA delivery is widely used tool to study cell biology and gene function in many plant species. This method relies on the ability of *A. tumefaciens* to transfer part of its DNA (T-DNA) to a plant cell and its incorporation into the plant genome. However, this system is not very effective for some cell types such as mcs due to a thick cell wall providing a physical barrier for the *Agrobacterium* transfer apparatus and demands additional penetration-enabling procedures. For example, Abdollahi et al. (2009) achieved transient transgene expression only when mcs cell walls were disrupted by microprojectile bombardment and then subjected to *Agrobacterium*-mediated transformation.

**Fig. 1:**
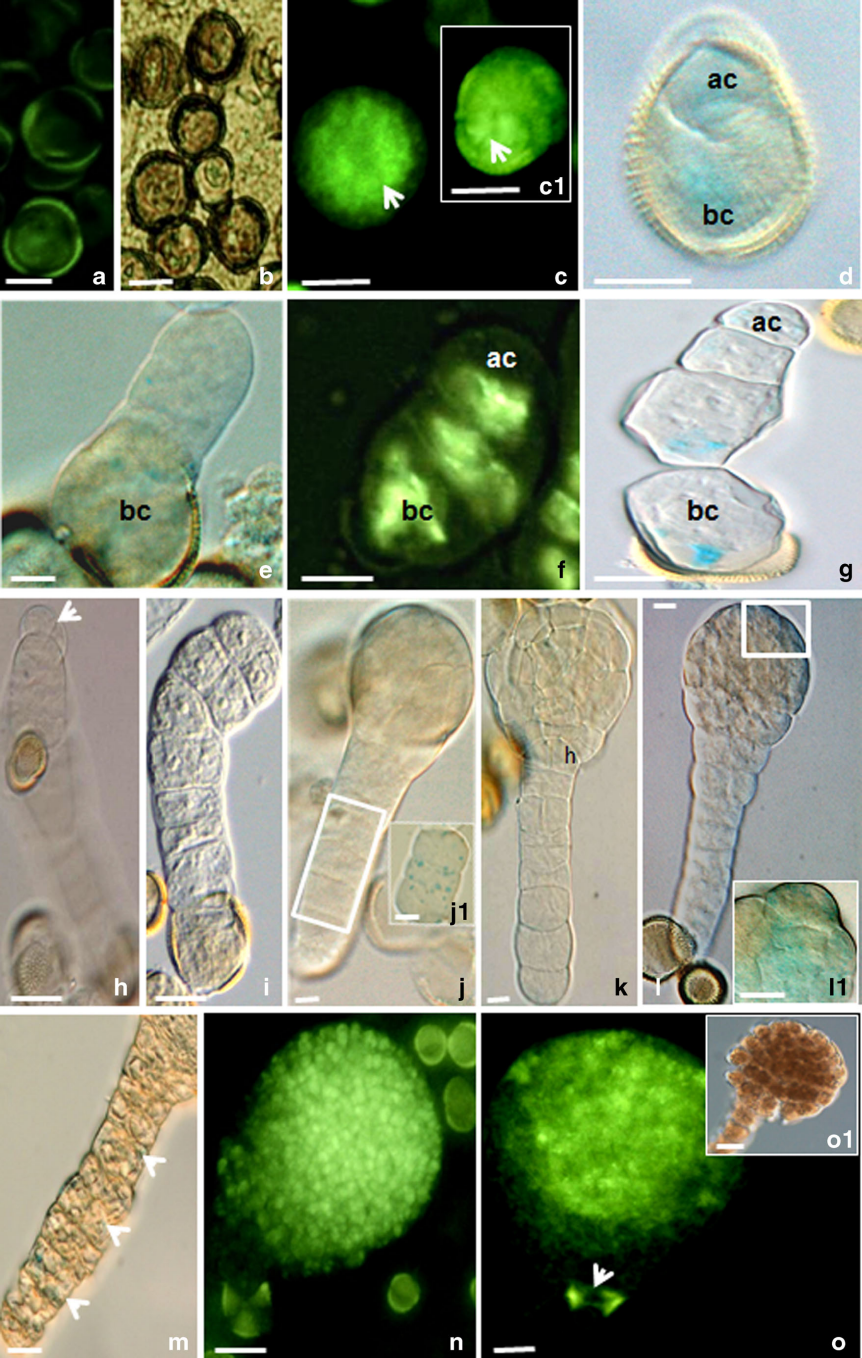
Local auxin distribution in the “mild” heat-treated microspores and MDEs of *B. napus.* **a** Uni-nucleated control microspores. Autofluorescence. **b** Uni-nucleated microspores 2 days co-cultured with *A. tumefaciens*. **c** -***c1*** Microspores with unequal auxin distribution on the one pole (*arrows*). **d** Two-celled pro-embryo with the basal (*bc*) and apical (*ac*) cells. **e**, **f** Linear file of three cells with the DR5 (**e**) or DR5rev (**f**) activities in all cells. **g** Linear file of four cells with higher DR5 activity in the basal cell (*bc*). **h**–**l** The pro-embryo proper with the suspensor-like structure. **h** Two-celled pro-embryo proper with a long suspensor-like filament. The tip cell of the suspensor-like structure was delineated to become the embryo proper after longitudinal division. **i** The embryo proper in the octant stage. **j** The globular embryo stage with the DR5 activity in the cells of the suspensor-like structure (*j1*). **k** The embryo proper in the dermatogen stage. Note the hypophysis region (**h**). **l** The globular embryo proper with maximum of the DR5 activity in the protoderm of the apical region of the embryo proper (*square*). The higher magnification of the region of protoderm with the DR5 activity on **l** (*l1*). **m** The DR5 activity in the cells of the suspensor-like structures at the globular stage embryo proper. **n** DR5rev activity in the apical part of the globular pro-embryo proper with suspensor. **o** Globular embryo proper with the suspensor. The DR5rev activity concentrated in the apical part of the pro-embryo, in the provasculature, and in the hypophysis (*arrow*). DIC image of the same representative as on **o** (*o1*). *Blue* and *light green colors* show the expression of the reporter *gus* and *gfp* genes driven by the DR5 or DR5rev promoters (respectively). *Bar* = 20 μm

**Fig. 2:**
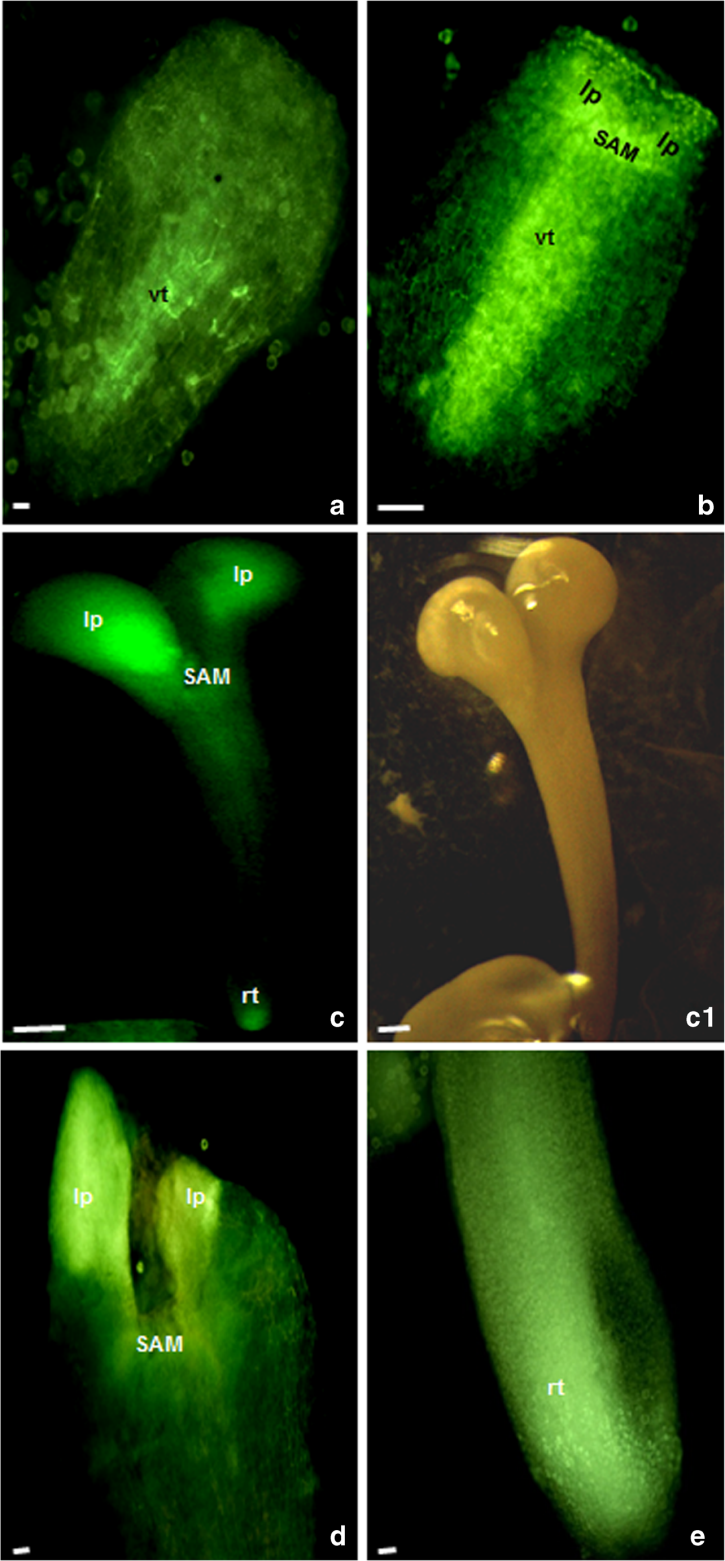
Local auxin distribution at the torpedo- and cotyledon-stage embryos of *B. napus* derived from the “mild” and prolonged heat treatment. **a**, **b** The torpedo-stage embryos. The DR5rev activity in the provasculature (*vt*), in the shoot apical meristem (*SAM*), and in the leaf primordia (*lp*). **c**–**e** The cotyledon-stage embryos with the DR5rev activity in the apex of cotyledons (*c*), in the shoot apical meristem (*SAM*), and in the root tip (*rt*). The *light green color* shows the expression of the reporter *gfp* gene driven by the DR5rev promoter. *Bar* = 20 μm

**Fig. 3:**
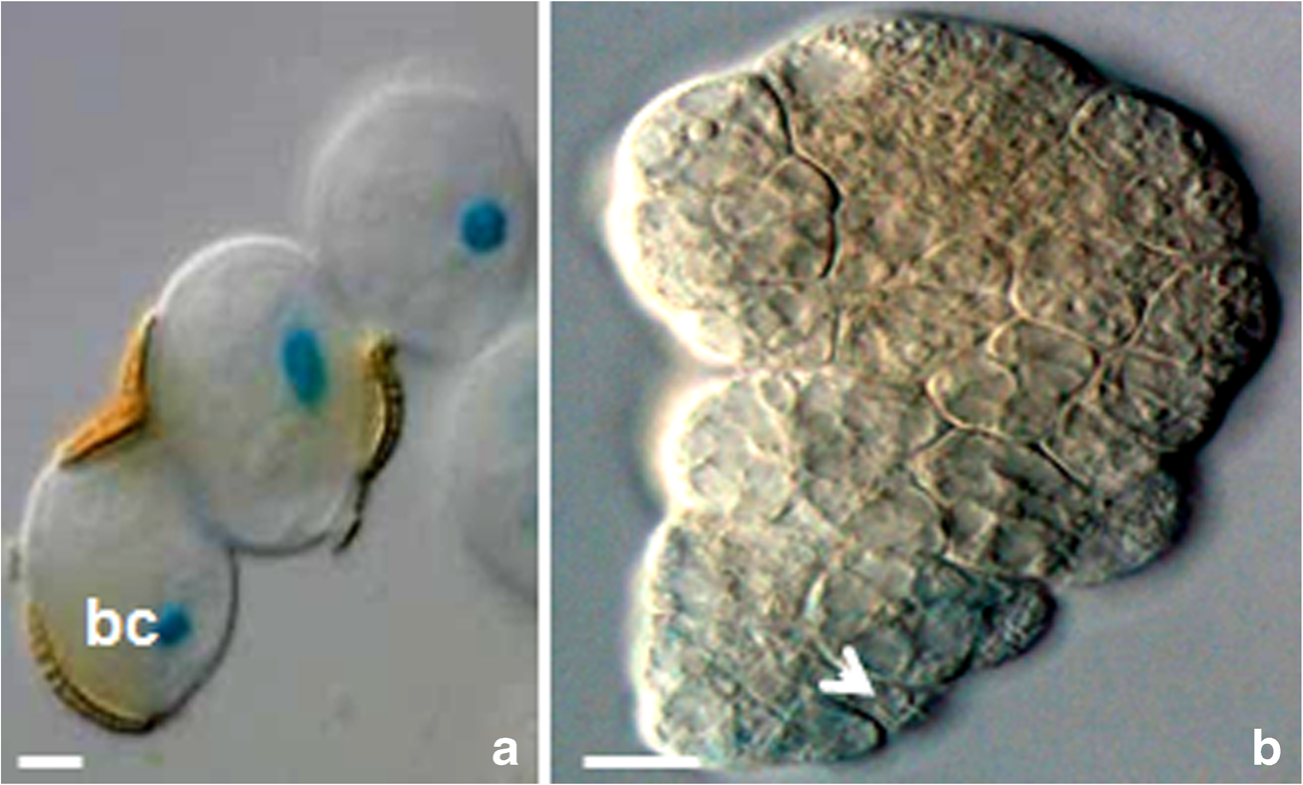
Local auxin distribution in the aberrant phenotypes of MDEs of *B. napus* derived from the “mild” heat treatment. **a** Three-celled callus-like structures consisting of loose cells with any polarity in the DR5 activity. The position of the basal cell (*bc*) remarkably marked dehiscent exine. **b** Mushroom-like embryo phenotype with the DR5 activity in a structure resembles “foot” of mushroom (*arrow*). The *blue color* shows the expression of the reporter *gus* gene under control of the DR5 promoter. *Bar* = 20 μm

**Fig. 4:**
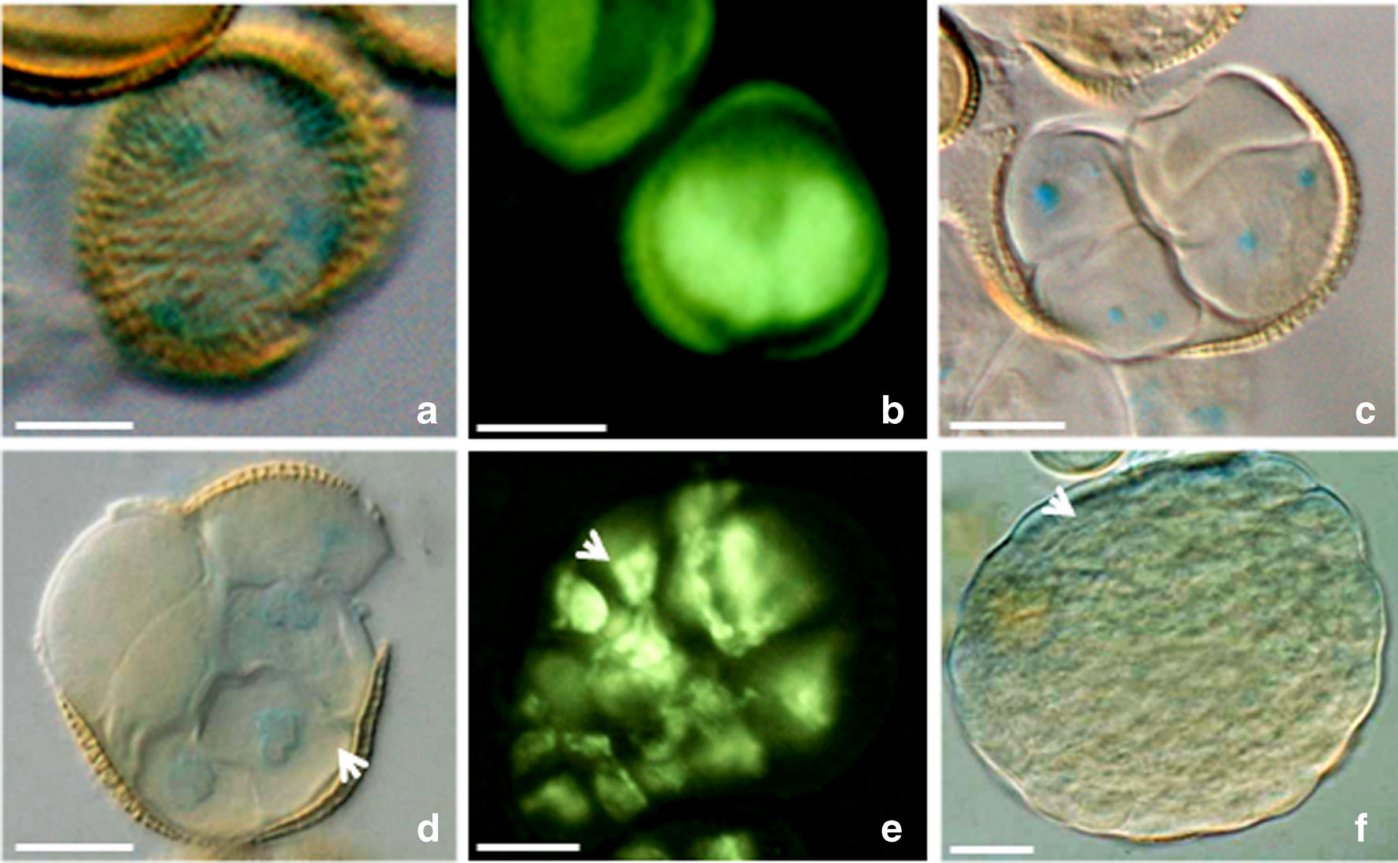
Local auxin distribution in the prolonged heat-treated microspores and MDEs of *B. napus.* **a** The microspore with non-polar activity of the DR5 promoter. **b** Two-celled pro-embryo with the DR5rev activity in the both symmetrical cells. **c**–**e** Multicellular structures emerging from the exine. The polar DR5 (**c**, **d**) or DR5rev (**e**) activities at the few-celled stage. The *arrow* indicates the stronger DR5rev activity on the one pole (**e**). (**f**) The globular embryo stage with the DR5 activity in the protoderm (*arrow*). The *blue* and *light green colors* show the expression of the reporter *gus* and *gfp* genes driven by the DR5 or DR5rev promoters (respectively). *Bar* = 20 μm

In the presented study, mcs were firstly induced to embryogenesis by the “mild” or prolonged heat treatment and then, 7 or 21 days post heat stress, subjected to the genetic modification procedure for 2 days. The T-DNAs contained the *gus* or *gfp* reporter genes under control of the auxin-responsive DR5 or DR5rev promoters (respectively). The local auxin distribution was demonstrated by the imaging reporter genes expressions (Figs. 1, 2, 3 and 4).

### DR5 and DR5rev promoter activities in the “mild” heat-treated mcs

In the uni-nucleated mcs, DR5rev promoter activity with stronger signal at one pole was detected in the majority of the “mild” heat-treated mcs (Fig. 1b, c [c1]). After slightly asymmetric transverse division, the DR5 activity was demonstrated in both apical and basal cells. However, higher activity was noticed in the basal cell (Fig. 1d), known as the place of auxin biosynthesis from which the hormone is transported to the apical cell. Established apical–basal polarity is one of the most important factors in zygotic as well as non-zygotic embryo formation (Mansfield and Briarty 1991; Liu et al. 1993; Laux and Jürgens 1997; Friml et al. 2003; Jenik et al. 2007; Supena et al. 2008; Dubas et al. 2011). Such restricted auxin localization at the only one cell of the young two-celled embryo could be required for the apical auxin response maximum and the specification of the apical embryogenic structures. This apical–basal auxin distribution could be translated to an activity gradient actively maintained by the novel components of auxin efflux such as PIN-FORMED (PIN) carriers (Friml et al. 2003; Supena et al. 2008).

When the suspensor-like structure was three or four cells long, the DR5 or DR5rev activities were observed in the apical cell and the cells below the apical cell (Fig. 1e–g). The basal cells of the suspensor-like structure were characterized by stronger signal (Fig. 1e–g). Following a series of transverse divisions, basal cell derivatives formed a uniseriate file of cells that resembled a typical suspensor of a zygotic embryo (Fig. 1h–l). Described above developmental pattern strongly suggests that directional auxin flow can take place from the suspensor basal cell and its derivatives to the apical cell. Suspensor-like structures could consist of up to eight cells before the apical cell divided longitudinally, which initiated an embryo proper formation (Fig. 1h–k). At the middle globular stage of the embryo proper, the DR5 or DR5rev activities were observed in the suspensor-like structure cells (Fig. 1j (j1)) as well as in the apical region of the embryo proper (Fig. 1l (l1),1n). At the dermatogen stage (Fig. 1k), the auxin was accumulated in the uppermost cell of the suspensor to form the hypophysis, the founder of the stem-cell niche of the embryonic root (Friml et al. 2003). Before embryo transition to the heart stage, strong DR5rev activity was detected at the places where the cotyledon primordium and provascular strands began to form (Fig. 1o). On the contrary, in the suspensor cells except for the region of the hypophysis, decreased DR5rev activity was detected (Fig. 1o).

The DR5rev activity was obviously enhanced at further developmental stages (Fig. 2). At the torpedo stage (Fig. 2a, b), the auxin was found in the center of the cotyledon primordia in the apical domain to establish the cotyledons. The DR5rev signal was positioned at the place where the shoot apical meristem (SAM) was probably formed (Weigel and Jürgens 2002). The strongest signal was observed in the leaf primordial and shoot apical meristems, and the provascular tissue (Fig. 2a, b). At the mature cotyledon stage (Fig. 2c–e), the DR5rev activity was observed in the apex of cotyledons, SAM (Fig. 2c, d), and the root tip (Fig. 2e).

Different DR5/DR5rev activities reflected the effect of the developmental stage on auxin maxima. Auxin gradients and concentration maxima within developing tissues provide positional cues for embryo differentiation and were probably determined by both auxin biosynthesis and auxin transport (Friml et al. 2003; Leyser 2006). Our observations are in agreement with data suggesting that distinct auxin sources provide a necessary trigger for the coordinated cell polarization, subsequent apical–basal axis orientation, and the axiality of the adult haploid plant determination (Robert et al. 2013).

In some cases (∼0.1–1 %), the embryos with abnormal phenotypes have been observed (Fig. 3). Such anomalous morphology was also accompanied by aberrant DR5 activity. No polarity in the DR5 activity was observed in the three-celled callus-like structures consisting of a number of loosely connected cells (Fig. 3a). Usually, the remains of the exine, located at the one pole, marked the polar growth of a callus-like structure. Recently, Tang et al. (2013) described that the first division plane of embryogenic mcs was induced by the position of the ruptured exine. In the “mushroom-like embryo” phenotypes, the DR5 activity was observed with its maximum in a structure resembling a “foot” of a mushroom (Fig. 3b). It resembles *A. thaliana pilz* mutants characterized by abnormalities in both the embryo proper and the suspensor. Such mutant suspensor has altered morphology and contains fewer cells than normal (Mayer et al. 1999).

### DR5 and DR5rev promoter activities in the prolonged heat-treated mcs

High DR5 expression was observed in the uni-nucleate microspore (Fig. 4a). However, after symmetric division, no polar DR5 expression was detected. Both, equally sized daughter cells, comprised the source of auxin (Fig. 4b). The DR5 expression began to differentiate at the early few-celled pro-embryo stage, when the exine ruptured (Fig. 4c, d).

When a multicellular structure was released from the exine, the higher DR5rev signal intensity with more intense was observed at the only one pole (Fig. 4e). This polar auxin distribution lasted up to the late dermatogen stage (Fig. 4f). These results probably not only are the effect of the heat duration but also reflect genome-regulated pattern of *B. napus* MDEs development. Such polar pattern of reporter gene activity can probably mark the apical–basal embryogenic axe formation (Fig. 4f).

### Estimation of endogenous auxin level

HPLC analysis together with the simultaneous measurements of the cells size, performed on the same cell suspension, allowed to estimate the mean concentration of IAA per single mcs of *B. napus*. In freshly isolated mcs, auxin concentration amounts to 0.2 μM when assuming a mean mcs’ radius of 10 μm. The “mild” heat stress (1 day at 32 °C) resulted in the increase of cell volume (mean radius 20 μm) and in the increase of auxin concentration to 1.01 μM. After prolonged heat treatment (5 days at 32 °C), when pro-embryos were at a few-celled stage, auxin content increased almost 100-fold in comparison with the auxin content detected at the single cell level (Dubas et al. *in preparation*). Such high auxin level could characterize dividing cells being stimulated to grow. As auxin is necessary for the progression of cell division, this finding indicates that this concentration of auxin could be necessary for the cell division initiation (Perrot-Rechenmann 2010).

### DR5rev promoter activities in the auxin-treated mcs

**Fig. 5:**
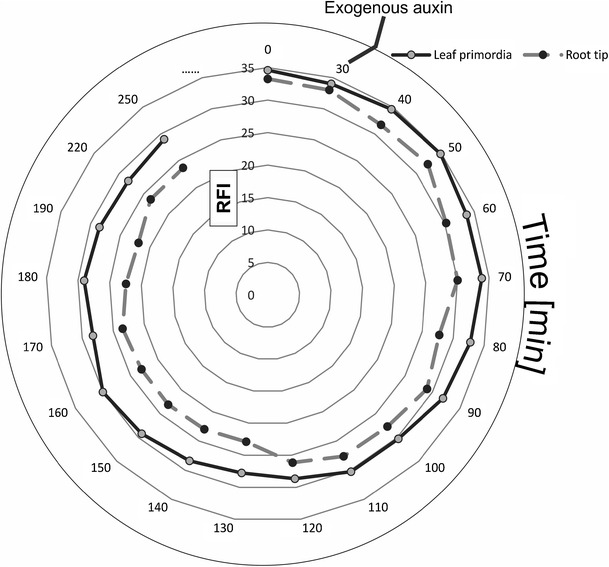
The relative fluorescence intensity (RFI) of DR5rev::GFP in MDEs exposed to the exogenous auxin in darkness. Application of IBA decreases the DR5rev::GFP fluorescence. DR5rev-GFP expression patterns in the leaf primordia and root tips at 10–30 min intervals over 220 min. Interval 0–30 min is dedicated for control (culture without the presence of exogenous auxin)

The shoot- and root-derived auxin pools participate in the control of plant development (Davies 2004). In order to determine the physiological response of the individual MDEs to applied exogenous auxin, the expression of GFP fluorescence was quantified on the basis of time-lapse imaging analysis. The relative fluorescence was fluctuating in a period of 220 min and changed in the range of 34.85–27.77 (max–min) in leaf primordia and 33.26–21.91 (max–min) in the root tips. Indole-3-butiric acid that induced decrease in the DR5:GFPrev fluorescence shows that IBA is probably able to influence the transcription of auxin-induced genes in embryonic leaf and root primordia. However, this effect was different from those usually observed in the case of IAA (Lewis et al. 2013). Unfortunately, there is no information available describing the mechanism of regulation of IBA response gene expression. Expected of an active auxin, the RFI intensity of primordia exposed to auxin was 1.31× higher of that measured in the root tips (Fig. 5). The data presented are in agreement with results received in classical auxin location experiments (Davies 2004). Quantitative estimation (spectrophotometric analysis) of auxin in the segments of plant seedlings revealed that the stem apex possesses 1.5- to 2.0-fold higher amounts than that found in the root apex. Although shoot apices synthesize more auxin than the root apices and both places of auxin production are critical for primordia, shoot, and root apical meristems formation and subsequent growth, we have noticed that the leaf primordia were more influenced by exogenous auxin than the roots in MDEs at the early stages.

Taken together, our results provide information that heat treatment does trigger auxin polarization in isolated microspores of *B. napus*. Auxin in concentration of 1.01 μM within the single cell correlates with unevenly expression of DR5/DR5rev. Unfortunately, there are little published data on the subcellular distribution of auxin in any plant species (Woodward and Bartel 2005). Regardless on the duration, the heat stress had a significant influence on the pattern of the auxin distribution at different developmental stages of the MDE. Moreover, after “mild” stress, the polarity appeared already in the microspore before the first endosporic cell division, which resulted in the embryos with suspensor-like structure formation. It seems that such auxin distribution gradient is necessary for the pattern of development that mimics almost perfectly zygotic embryo formation *in planta*. By an analogy, it could be assumed that the auxin gradient plays also an important role from the earliest stages of zygotic embryogenesis. In some cases, the formation of mushroom-like structures or loosely connected cells was observed. The presence of such aberrant phenotypes suggests the functional importance of the auxin directional flow within the suspensor. In the prolonged heat-treated mcs, the establishment of pro-embryo polarity was determined after symmetric endosporic divisions of the few-celled embryo and coincided with the exine rupture. It is possible that auxin, as a main polarity marker, induces the moment of exine rupture by what finally defines embryogenic polar growth. Our results are contradictory with the data of Tang et al. (2013), who postulated that exine dehiscing induces rape microspore polarity and this polarity results in a different cell fate and fixes the apical–basal axis of embryos on NLN-13 medium supplemented with mannitol.

With the use of the unique *B. napus* microspore suspension system, we were able to present the switch from the symmetrical to asymmetrical auxin distribution dependent on the heat stress duration and its consequences in the pathways of MDEs development. Moreover, we determined the endogenous auxin concentration in a single cell that is suitable for embryogenesis initiation. The presented system can be also used in studies examining the effects of the various exogenously applied substances on the endogenous auxin level.

## Abbreviations

*Aux/IAA*: *Arabidopsis thaliana* gene family immediately induced by auxin
AuxRE: The consensus TGTCT sequence within the promoter region of the auxin-responsive genes
*GH3*: *Arabidopsis thaliana* gene family immediately induced by auxin
*SAUR*: *Arabidopsis thaliana* gene family immediately induced by auxin
DR5: The synthetic auxin-responsive promoter
DR5rev: The synthetic auxin-responsive promoter
*gus*: β-Glucuronidase reporter gene
*gfp*: Green fluorescent protein marker gene
GV3101: *Agrobacterium tumefaciens* strain
IAA: Indole-3-acetic acid
IBA: Indole-3-butyric acid
LBA 4404: *Agrobacterium tumefaciens* strain
mcs: Microspores
MDEs: Microspore-derived embryos
NLN-13: A liquid medium with 13 % sucrose
OD: Optical density
*pilz*: *Arabidopsis thaliana* mutants
PIN: PIN-FORMED carrier proteins
RFI: The relative fluorescence intensity
SAM: Shoot apical meristem
T-DNA: The transfer DNA
X-Gluc: 5-Bromo-4-chloro-3-indolyl-β-D-glucuronide
